# Are Online Maps and Booking Systems for Antenatal Vaccination Fit for Purpose? A Qualitative Study

**DOI:** 10.3390/vaccines13121202

**Published:** 2025-11-28

**Authors:** Paepa Tohaia, Amber Young, Esther Willing, Louise Fangupo, Gabrielle McDonald

**Affiliations:** 1 Kōhatu – Centre for Hauora Māori, Faculty of Medicine – Dunedin, Division of Health Sciences, University of Otago, Dunedin 9016, New Zealand; tohpa215@student.otago.ac.nz (P.T.); esther.willing@otago.ac.nz (E.W.); 2 School of Pharmacy, Faculty of Health Professional Programmes, Division of Health Sciences, University of Otago, Dunedin 9016, New Zealand; amber.young@otago.ac.nz (A.Y.);

**Keywords:** maternal vaccinations, antenatal vaccinations, Māori health, healthcare inequities, health websites

## Abstract

**Objective**: Online maps and booking tools aim to reduce barriers to vaccination by helping users locate nearby clinics, understand service availability, and provide information about vaccination choices. The aim of this research was to explore the potential of online vaccination booking and mapping tools in facilitating antenatal vaccination uptake among Māori and Pacific hapū māmā (pregnant women) in Aotearoa. **Methods**: This was a qualitative descriptive study that used kaupapa Māori methodology. Focus groups with hapū māmā and semi-structured interviews with healthcare workers were conducted. Transcripts were analyzed thematically. **Results**: Seven hapū māmā and forty healthcare professionals participated. Three main themes were developed from interviews with hapū māmā: (i) limited awareness of online maps and bookings for vaccination services; (ii) the need for accessible, user-friendly information; and (iii) preference for flexible booking systems. Three themes were developed from the healthcare worker interviews: (i) difficulties with accessibility; (ii) promotion of tools; and (iii) usability of online platforms. **Discussion**: Although antenatal vaccinations are publicly funded, systemic and digital barriers persist, especially for Māori and Pacific communities. Online tools have the potential to support maternal vaccination, but require improved visibility, cultural relevance, and functionality to be effective.

## 1. Introduction

Antenatal vaccinations are essential for safeguarding hapū māmā (pregnant women) and their pēpi (babies) against severe vaccine-preventable diseases such as pertussis and influenza [1]. Vaccination during pregnancy provides passive immunity for pēpi through antibodies transferred via the placenta and breast milk, protecting pēpi until their next recommended vaccinations at six weeks (pertussis) and six months (influenza) [2].

Influenza infection during pregnancy increases risks of preterm birth, low birth weight, and stillbirth [3], as well as causing increased morbidity and mortality in hapū māmā [4]. Pertussis in pēpi can lead to severe complications, including death [5]. Antenatal influenza vaccination has been shown to reduce adverse outcomes associated with infection, with a significant reduction in both laboratory-confirmed influenza cases and hospitalization of babies of vaccinated mothers [6]. Antenatal pertussis vaccination is estimated to be over 90% effective at reducing the number of babies hospitalized from pertussis infection and 94% effective at preventing death [7]. These benefits underscore the importance of all pregnant women being vaccinated.

In Aotearoa (New Zealand), antenatal vaccination for influenza and pertussis has been fully funded since 2010 and 2013, respectively [2]. These are the only vaccinations that are currently recommended for all pregnant women. Influenza vaccination is recommended at any stage of pregnancy, but is best at the start of the influenza season, while pertussis vaccination (Tdap) is advised from 16 weeks’ gestation of every pregnancy, but ideally in the second trimester or later, and at least two weeks prior to the birth of the baby [8]. Despite these recommendations, a retrospective study in Aotearoa showed that in 2020, only 43.6% of hapū māmā were immunized for influenza and 48.0% for pertussis [9].

Significant disparities in antenatal vaccination rates exist for Māori and Pacific communities in Aotearoa, reflecting structural inequities within the healthcare system [10]. Pākehā (New Zealander of European descent) women are more likely to access and receive appropriate vaccinations during pregnancy, while Māori and Pacific people face substantial systemic barriers [11]. Howe et al. [12] found that wāhine Māori are less likely to be vaccinated against pertussis (OR 0.55) and influenza (OR 0.69) compared with New Zealand European or non-Māori women.

These disparities reflect structural privilege, where access and opportunities disproportionately favor Pākehā women [10]. The historical and ongoing prioritization of the dominant culture within the healthcare system leads to adverse health outcomes [2,10]. For example, pēpi Māori have pertussis infection rates 2.5 times higher than other ethnic groups during their first year of life.

Addressing disparities requires recognizing and dismantling structural privilege while ensuring equitable access to vaccination services for all hapū māmā. Barriers to antenatal vaccination include limited access to lead maternity carers (LMCs), transportation challenges, financial constraints, caregiving responsibilities, and a lack of culturally appropriate care [13]. Misinformation and poor communication about vaccine safety further exacerbate these challenges [14,15,16,17]. Culturally tailored approaches (in our case, integrating tikanga Māori (Māori protocols and practices)) and addressing logistical challenges are essential to improving vaccination uptake [17].

There is a need for multiple interventions to support increased vaccination coverage, including the use of digital technology to streamline the vaccination process [18,19]. Online maps showing where to access vaccination services, and online appointment booking tools have the potential to address some barriers to vaccination uptake by providing clear, accessible information about clinic locations, hours, costs, availability, and accessibility. Booking tools can also provide an easy way to schedule an appointment at a location and time that suits the individual, allowing them to schedule appointments around work, childcare, or other commitments [20,21]. In Aotearoa, there are online vaccine appointment booking tools and location maps freely available. The appointment booking tool was originally developed during the COVID-19 pandemic but has since widened in functionality to include booking for other vaccinations, including antenatal pertussis and influenza vaccines. Although everyone in Aotearoa can visit the websites that have locations of vaccination venues and online booking tools, it is not known if these tools are known about, utilized, or perceived to be usable by hapū māmā, and whether there are opportunities to increase the utility of such tools.

This study explored healthcare professionals and hapū māmā opinions on (i) whether online vaccination maps and booking tools meet the needs of hapū māmā in Aotearoa; (ii) if maps and booking tools have the potential to support equitable antenatal vaccination uptake; and (iii) how the maps and booking tools could be improved to make them more effective.

## 2. Materials and Methods

### 2.1. Research Design

This qualitative study employed kaupapa Māori methodology (research by Māori, for Māori and with Māori) [22,23] to explore the potential of interventions to improve antenatal vaccination uptake for Māori and Pacific hapū māmā. This study was conducted within a larger antenatal vaccination research study investigating several interventions to increase immunization uptake in pregnancy [24]; online maps and booking tools were one component of the interventions investigated. Two online vaccination booking systems that are available throughout Aotearoa that show a range of providers and include maps are: Bookmyvaccine (https://app.bookmyvaccine.health.nz/) (URL accessed 3 April 2025) and Healthpoint (https://www.healthpoint.co.nz/) (URL accessed 3 April 2025). These two platforms were used as examples if participants required prompts.

There were two arms to this study: (i) focus groups and interviews (depending on participant availability) with hapū māmā (n = 7), and (ii) interviews with healthcare workers (n = 40). This sample size was selected based on similar research [11,25,26,27] and concepts of information power described by Malterud et al. [28]. Māori and Pacific voices were intentionally prioritized through recruiting only Māori and Pacific hapū māmā, and by ensuring substantial representation of Māori and Pacific healthcare professionals. Ethical approval was obtained from the University of Otago Human Ethics Committee (H23/113) and Māori research consultation was undertaken with the Ngāi Tahu Research Consultation Committee (24199). A semi-structured interview guide for hapū māmā was developed based on the literature, with input from the research team, which included healthcare research academics, academic GPs, a Māori academic advisor, an epidemiologist, a midwife, and a pharmacist. The guide was developed to determine the level of knowledge of current online resources, how the online resources could be improved to make them more effective, and what barriers and enablers participants could identify for using the resources. This was piloted with a recently hapū māmā, and modifications were made following the pilot.

An interview guide was developed for the healthcare workforce that would explore awareness of online booking systems and how they are currently used and recommended to hapū māmā, as well as what they thought worked well, and their ideas for improvements that could be made. A pilot of the interview questions was undertaken with two healthcare professionals. Following the pilot, questions were refined to ensure that important aspects were focused on and to reduce duplication of questions.

### 2.2. Sample Strategy

#### 2.2.1. Hapū Māmā

Focus groups were conducted in Porirua and Gisborne. These sites were selected because they have high proportions of Māori and Pacific people and known disparities in antenatal vaccination rates. Recruitment was facilitated by community health organizations Ora Toa (Porirua) and Tūranga Health (Gisborne), using clinical contacts and antenatal classes. Participants were purposively sampled to meet our inclusion criteria, which were that māmā must be hapū, identify with Māori or Pacific ethnic groups, be aged at least 18 years old, and be literate in spoken English.

#### 2.2.2. Healthcare Professionals

Healthcare professionals from a range of practice backgrounds, including practice nurses, general practitioners, midwives, pharmacists, vaccination coordinators, kaiāwhina (non-regulated healthcare workers), and Māori and Pacific health providers, were recruited as part of the larger antenatal vaccination research study. Recruitment was facilitated by professional networks, newsletters, and snowballing techniques. Workers had to be employed and working in vaccination and primary healthcare in Aotearoa and be literate in English to be eligible to participate in the study.

### 2.3. Data Collection

At the start of each focus group/interview, informed consent was obtained, and a short demographic questionnaire was filled out by participants, including, for healthcare workers, their role. Sessions were recorded and transcribed verbatim using TurboScribe (TurboScribe.ai Inc., USA), (https://turboscribe.ai/ accessed 11 November 2024) which were then checked against recordings. Transcripts were securely stored in an electronic folder on an onshore server, as part of our commitment to Māori data sovereignty [29].

#### 2.3.1. Hapū Māmā

Focus groups lasting approximately two hours were conducted kanohi ki te kanohi (face-to-face). As part of our kaupapa Māori methodology, whakawhanaungatanga (relationship-building) preceded discussions to support open conversations. Grounding the dialogue within this cultural setting ensured that participants were all able to have their voice heard and helped people to build respect and understanding with each other before the beginning of data collection. We also practiced manaakitanga (hospitality and generosity) with participants by providing koha ($100 voucher) and kai (food) during the focus group/interview sessions. See Appendix A for focus group outline.

#### 2.3.2. Healthcare Professionals

Interviews were semi-structured and lasted around an hour (Appendix B). Participants were offered a $50 voucher as a thank you for their time.

### 2.4. Research Characteristics and Reflexivity

As a medical student with whakapapa (ancestry) to Ngāti Raukawa, PT’s commitment to supporting Māori communities drives this research. Addressing antenatal vaccination inequities aligns with her career goals of improving health outcomes for Māori. Other members of the research group consisted of healthcare professionals (public health physician, pharmacist, dietician) and Māori health researchers. All researchers involved in the project, a mix of Māori and Pākehā, support equitable antenatal vaccination opportunities for Māori and Pacific hapū māmā.

### 2.5. Analysis

A kaupapa Māori lens was used in the interpretation of findings. This reflected our study values of whānau ora (wellbeing of family as a group), whakapapa (ancestry), manaakitanga (generosity and support) and tino rangatiratanga (self-determination). Data were analyzed using directed qualitative content analysis [30,31,32], facilitated by NVivo software (Lumivero, Denver, CO, USA; version 12). Transcripts were read and reread multiple times to enable familiarity with the data and generate codes. Codes were grouped into themes and then regrouped as the analysis progressed. Codes and themes were reviewed by another author.

## 3. Results

Three focus groups and one interview were conducted, each targeting four participants. Two shorter focus groups in one community were on consecutive days, with the same four participants on both days. The first focus group discussed the booking tools and online maps and investigated participants’ opinions. The second focus group explored participants’ opinions on how they should be designed and ways to reduce barriers to their use. In the other community, the focus groups were combined into one longer session to be held in a single day for acceptability purposes for participants. One focus group had two participants and although a second was planned, some participants were unable to attend on the day. As the other participant of that focus group had already arrived, the decision was made to proceed with the same questions, but to conduct the session as an interview. All participants identified with Māori and/or Pacific ethnic groups. Forty health professionals were interviewed. Interviews were conducted either kanohi ki te kanohi (face to face) in Dunedin or Gisborne, or by Zoom (San Jose, CA, USA, version 6.6.10). Health professionals came from 12 locations throughout Aotearoa. Half (n = 20) of the healthcare professionals interviewed identified as either Māori or Pacific ethnicity.

### 3.1. Hapū Māmā

Hapū māmā provided insight into the utility and barriers of online vaccination mapping tools in supporting equitable vaccination uptake. The demographic characteristics of hapū māmā who participated in the research are presented in Table 1. Three key themes were derived from the data: awareness of online vaccination services, information accessibility, and preferences for interaction and booking methods.

### 3.2. Awareness of Online Vaccination Services

Limited awareness of online booking systems and maps showing vaccination services was identified as a major barrier, with many participants reporting minimal experience with these resources. The data showed that hapū māmā often only became aware of such services through incidental exposure, such as employment or social networks. Most hapū māmā in the study were not aware of existing online vaccination maps and booking systems. One participant who was not a healthcare worker but had employment in a healthcare setting commented that her work environment was the only reason she had knowledge of these resources.

“Since I work at [a health organization], that is how I became aware of the [online platform for] vaccinations because I work there, and it is always talked about and stuff like that. But I think if I didn’t work there, then I probably wouldn’t be aware of that.”

(HM03)

Although platforms like Bookmyvaccine and Healthpoint gained prominence during the COVID-19 pandemic for COVID-19 vaccination, participants were unaware that they were able to access antenatal vaccination locations and bookings through these sites.

“If you type into Google, where can you go to get vaccinated, would that pop up?”

(HM03)

Participants viewed midwives as a trusted source of information. However, midwives rarely provided information about online maps and booking tools for vaccination services. When asked if they thought their midwife could use these maps or websites when talking to hapū māmā, participants thought they could, although they noted that midwives often waited for them to initiate discussion about vaccination and took a more passive approach.

“I think my midwife would just wait for me to ask, like, “Hey, you know where I can get vaccinated?” And she would pull it up and be like, “Here, here, and here,” or she will tell me to have a look at these sites … It is more of a wait until you seem open to it kind of thing, rather than a forced hand for it.”

(HM02)

Participants also suggested targeted advertising for maps and booking systems as an effective way to raise awareness, particularly on social media platforms. Unskippable online videos, television campaigns, and other persistent reminders were recommended to ensure the message of online services reaches hapū māmā in a relatable and unavoidable manner:

“Those little ads that come up … make those ones unskippable.”

(HM02)

### 3.3. Information Accessibility

The accessibility and functionality of information provided on online mapping tools were identified as important. Hapū māmā emphasized that tools should offer clear, detailed, user-friendly, up-to-date information, and have practical features to support informed decision-making:

“If I am already trying to look on where to go, I want to know what time it is open, what days it is open … because if it is a pharmacy, there might be times where ‘We don’t have anyone [available to vaccinate] today.’”

(HM02)

Participants also stressed the importance of additional features for online maps to better accommodate the diverse needs of hapū māmā, including places with after-hours service availability for individuals with demanding schedules.

“That would be good if there was, like, an after-hours [option].”

(HM05)

Navigation challenges were identified as recurring barriers for hapū māmā. Frustration was expressed among participants about generic GPS instructions with maps on websites that failed to pinpoint exact locations or landmarks:

“When you do Google Map and you are outside five different shops, it just says, ‘You’ve arrived!’ Oh mate!”

(HM03)

These challenges not only create inconvenience but may also discourage some hapū māmā from using online mapping tools and booking systems altogether. To address these issues, participants recommended incorporating visual aids such as pictures of storefronts and street views to enhance usability and reduce confusion and navigation barriers:

“Click on said pinpoint and it opens a photo to the storefront.”

(HM02)

This would allow users to identify locations visually rather than depending solely on addresses. Dual functionality in map design was identified as being important. Having both interactive map visuals and a straightforward list of addresses was suggested to cater to varied user preferences and to improve accessibility.

### 3.4. Preferences for Interactions and Booking Methods

The third theme related to hapū māmā preference for accessing and booking vaccination services online. Participants were asked if they would rather book online or telephone vaccination services to make an appointment. A range of preferences was expressed, reflecting the importance of flexibility in booking methods. Many favored online booking systems for their convenience and efficiency, particularly those who preferred avoiding direct phone communication:

“Me personally, I just don’t like talking on the phone.”

(HM04)

For individuals who prefer less personal interaction, online tools offer an accessible and low-pressure alternative. Others described a preference for a hybrid approach, combining online tools for initial research with phone calls for confirmation:

“I would probably research it on the map and then find the phone number for that place and call them.”

(HM01)

Phone calls were favored for the immediacy they offer and the ability to find out more information if needed.

“I like to go straight to the source. Like, tell me what I need to know now.”

(HM03)

Flexibility in booking methods features consistently, and includes the ability to have options where booking isn’t necessary. Walk-in options were identified as essential, providing accessibility for individuals who may face barriers to online or phone-based interactions.

“Not restricting it to anything makes it easier for people to choose what works for them.”

(HM03)

### 3.5. Healthcare Professionals

The opinions expressed by hapū māmā on the benefits and challenges of online vaccination mapping tools in promoting equitable vaccination uptake were reinforced by interviews with forty healthcare workers. The demographic characteristics of healthcare professionals who participated in the interviews are presented in Table 2. Three main themes were identified: difficulties with accessibility; awareness and promotion of tools; and usability and clarity of online platforms.

### 3.6. Difficulties with Accessibility

Inequitable access to vaccination resources was identified as a significant barrier due to technological, financial, and infrastructure constraints, with concerns regarding limited internet access, device availability, and data affordability, which hinder engagement with online health resources.

“You can’t assume everyone’s got access to the internet.”

(HP15)

Healthcare workers also noted the high cost of technology for some users.

“We assume that most households have an iPad, computer, smartphones … not every whānau [family] have got the money to have an iPad and an iPhone, and they cost a lot of money.”

(HP16)

Workers expressed the need for alternative solutions, such as offline resources or printed materials, to ensure accessibility for individuals without reliable internet or digital devices.

“Not everyone’s got access to the internet, so maybe the midwife could print out a map.”

(HP15)

It was indicated that online maps may not support equitable access.

“You have to have a certain element of privilege to access online maps.”

(HP12)

Data-neutral options were also recommended to reduce challenges for those with limited data plans.

“Wouldn’t it be nice if it just worked and you didn’t have data?”

(HP14)

Limited digital literacy was another barrier, with some individuals struggling to navigate online tools.

“This takes a lot of knowledge to be able to look at the online maps, interpret the map, work out how to get there.”

(HP12)

### 3.7. Awareness and Promotion of Tools

A critical barrier to the uptake and utilization of healthcare resources identified was the inadequate promotion and consequent lack of awareness of existing tools among both healthcare workers and hapū māmā. Like hapū māmā, many healthcare workers admitted to being unaware of specific tools like Bookmyvaccine and HealthPoint, indicating gaps in communication and training.

“I knew Bookmyvaccine was around for COVID vaccines, which maybe is saying something … a health professional, don’t know how the resources work.”

(HP41)

Or if they were aware, they did not think to use them when talking about vaccination in pregnancy.

“I don’t think I’ve ever recommended to anybody, ever. And that’s for all across vaccines.”

(HP50)

Poor advertising and a lack of visibility were highlighted as barriers to people knowing about existing tools and in reaching hapū māmā. This lack of awareness and promotion not only limits resource utilization but also exacerbates inequities, as individuals unaware of available services are less likely to seek care. A need for more specific education for healthcare providers was voiced, making healthcare providers more equipped to guide patients more effectively through antenatal care.

“If they don’t know about it, then they won’t use it, so making sure that everybody’s aware that it is there if they need it.”

(HP26)

### 3.8. Usability and Clarity of Online Platforms

The complexity and inefficiency of current online platforms emerged as significant usability barriers. Participants consistently called for simplicity and clarity in design to improve accessibility for a broader audience, particularly for those with limited digital literacy or time constraints.

“It’s overcomplicated, it’s too hard. It should just be a map that actually you click. The search engine is way too complex. Even I struggle to find where I should be … It’s overwhelming in its information.”

(HP39)

Many also noted that the information on these platforms was sometimes out of date, making it difficult to rely on them for accurate information.

“Half the time it [the website] doesn’t get updated. It’s not accurate, it’s not updated as frequently as it should be.”

(HP39)

Online maps often posed challenges for healthcare workers who were unfamiliar with such tools.

“When I tried to use it, there was a lot of information there. It felt clumsy and difficult.”

(HP44)

Some healthcare workers from smaller communities highlighted that an online map was less necessary in their context, as healthcare providers already knew where to refer people for vaccinations.

“We’re a small community, so everyone knows anyway. So, it probably wouldn’t be hugely beneficial for us because we know where to go … And most people know where to go. Other health, like, you know, the pharmacists, the GPs, they know where to send everyone because we’re small.”

(HP43)

Participants suggested that tools should include essential practice details while minimizing unnecessary complexity.

“Just needs to show you where to go. Don’t overcomplicate it.”

(HP14)

## 4. Discussion

This study identified barriers to the use of online maps and vaccination booking tools for antenatal vaccination uptake, highlighting limited awareness and usability challenges in existing digital tools as well as suggesting systemic inequities in access to digital resources. Some hapū māmā reported learning about online tools incidentally, emphasizing gaps in proactive communication. Similarly, healthcare workers noted a lack of their own awareness and promotion of existing tools, with many admitting they were either unaware of resources such as Bookmyvaccine or Healthpoint, or unaware that they could be used for antenatal vaccination as well as for COVID-19 vaccination.

In our study, some hapū māmā reported that their midwives had not actively provided information about where to get vaccinated, despite online maps of vaccination venues and online booking tools being available. A reluctance to proactively discuss vaccination venues may be related to wider concerns about respecting autonomy and avoiding perceived coercion [13,33,34]. These tensions need to be managed, as proactive and clear information from midwives and LMCs about current tools has the potential to significantly improve antenatal vaccination rates, in part because patients are more likely to receive care that is recommended by a trusted provider [11,13,35].

Hapū māmā and healthcare professionals recommended various enhancements to optimize the current online tools. This is consistent with existing literature [36] showing that visibility improvements, such as better search engine optimization (SEO), significantly enhance user access and engagement with websites. Usability improvements, including providing after-hours service information, visual aids like clinic photos, providing clear directions to venues, and ensuring the information is up to date, were recommended by our study participants, consistent with known usability heuristics [37]. Additionally, targeted social media campaigns using forced-view (“unskippable”) ads, which were suggested to raise awareness and promote existing tools, have been demonstrated in recent studies to increase recall and message retention compared to skippable ads [38].

Participants in this study thought that online advertising, online maps, and booking tools would raise awareness of their availability and increase engagement. It has been demonstrated that social media can have a large impact on creating awareness [39], so using it for this purpose could achieve increased knowledge of these tools in Aotearoa. However, other research has identified that some people mistrust information online, so it might be that online advertising needs to be shared through trusted sources [40]. If agencies that create online booking tools and maps wish to advertise online, they may have a wider impact if they engage with trusted health providers across the country for support with dissemination to communities.

Healthcare workers identified significant accessibility challenges disproportionately affecting Indigenous and minoritized groups, including Māori and Pacific communities and those from low socioeconomic backgrounds. Barriers included limited internet access, high costs of technology, and unaffordable data plans, which hinder engagement with digital tools. Participants also described how poorly designed and difficult-to-use online tools were off-putting. These findings are consistent with other research examining digital health interventions amongst diverse populations [41,42,43]. This highlights the need for new approaches to promote equitable outcomes, incorporating offline alternatives such as printed maps or data-neutral solutions to support those without digital devices or reliable internet access. Additionally, limited digital literacy further compounds these challenges, emphasizing the importance of simplifying tools and providing clear, user-friendly guidance to improve accessibility for all [44].

### Strengths and Limitations

This study builds on research showing significant barriers to antenatal vaccination in Aotearoa, including structural inequities and the importance of culturally responsive healthcare practices [10,11,12]. While earlier studies have focused on healthcare provider roles and general strategies to improve vaccine uptake, this research uniquely examines the potential of digital tools, an area that has not been explored in the context of hapū māmā. Whilst digital tools have been found to improve vaccination rates [45], no prior studies have specifically examined the usefulness of online maps and booking systems for antenatal vaccination in hapū māmā. This study makes a unique contribution by identifying issues with current digital tools and offering culturally informed recommendations to improve them.

Other strengths of this study include the prioritization of the voices of these specific communities and populations and the alignment with the principles of kaupapa Māori research, which is research conducted by Māori, for Māori, with Māori [22,46].

Our sample size was smaller than anticipated, in part due to participants being unable to attend on the day. This required us to conduct one focus group as an interview, as we did not wish to inconvenience the remaining participant in that focus group. This was a pragmatic decision that is consistent with the study value of manaakitanga and was appropriate in this setting of kaupapa Māori research. Despite the sample size, it was suitable for qualitative research and the findings aligned with those from the healthcare professional interviews and gave sufficient data to achieve the study aims [28]. The recruitment of Māori and Pacific hapū māmā into studies can be difficult due to justified mistrust in mainstream systems [35,47,48]. Smith argued that “Research has historically marginalized Indigenous perspectives, often presented through an ‘imperial gaze.’” [49]. However, with our purposive sampling methods and partnering with Māori and Pacific healthcare organizations for this research, we managed to achieve good representation of Indigenous and minoritized Pacific people in our study.

Although we purposively sampled for Māori and Pacific hapū māmā to be recruited for this study, due to limitations in time and funding, we could only recruit those who could converse in English. This may have led to bias for those with barriers to accessing healthcare. Further work is necessary to investigate the needs of non-English speaking people residing in Aotearoa, and the needs of those who are more comfortable conversing in languages other than English.

It is clear that participants would like to use well-designed, easy-to-use online tools that contain up-to-date information. Quantitative research to examine how such tools impact on the reduction of barriers to vaccination would be of benefit to augment our exploratory qualitative study.

## 5. Conclusions

This study highlights the potential of booking tools and online maps showing vaccination venues to improve antenatal vaccination rates for hapū māmā. Many pregnant people remain unaware of existing online tools that could facilitate access to and uptake of vaccination services. Healthcare workers often fail to promote these resources effectively. Furthermore, existing digital tools lack essential practical details, such as up-to-date operational information and easy interfaces, all of which hinder usability.

Our exploratory study suggests that there are measures that could be taken to improve the usage of online maps and booking systems for vaccination. These include improving the usability of existing tools by adding practical information such as clinic hours, costs (or an explicit statement that the service is free), parking availability, accessibility features (e.g., large font sizes, translation options), visual aids (e.g., photos of clinics, clear directions), and offering multiple booking methods (online, phone, and walk-in). The visibility of tools by actively promoting the benefits and availability of online tools to Māori and Pacific hapū māmā through integrating online tool use in clinical care and conducting social media campaigns is also likely to be of benefit.

## Figures and Tables

**Table 1:** Demographic characteristics of hapū māmā (n = 7).

Characteristic	Number
**Ethnicity** *	
Māori	5
Samoan	2
Cook island	1
NZ European	1
**Languages spoken in addition to English ^†^**	
Te Reo (Māori)	2
Samoan	1
**Weeks pregnant**	
14–27	2
28–35	3
More than 36 weeks	2
**Age group (years)**	
18–29	4
30–39	2
40–49	1
**Currently have children**	
No	5
Yes	2
**Highest level of education**	
High school	2
Level 1–4 certificate	2
Level 5–6 certificate or diploma	1
Bachelor’s degree, graduate diploma/certificate	2
**Currently in paid employment**	
Yes	5
No	2

* Multiple answers were allowed; hence, totals could amount to >7. ^†^ Multiple answers were allowed. However, not all hapū māmā spoke two or more languages; hence, the total does not amount to 7.

**Table 2:** Demographic characteristics of healthcare professionals (n = 40).

Characteristic	Number (%)
Ethnicity *	
Māori	15 (37.5)
Pacific	5 (12.5)
British, Irish	3 (7.5)
New Zealand European	28 (70.0)
Other	3 (7.5)
**Languages spoken in addition to English ^†^**	
Te Reo (Māori)	4 (10.0)
A Pacific language	2 (5.0)
Other	1 (2.5)
Missing	4 (10.0)
Role *	
General Practitioner	2 (5.0)
Nurse	14 (35.0)
Pharmacist	3 (7.5)
Midwife	13 (32.5)
Immunization Coordinator/Advisor/Manager	6 (15.0)
Kaiāwhina ^‡^	3 (7.5)
**Employer**	
Non-Māori health provider or partnership	26 (65.0)
Māori health provider or Iwi Māori Partnership Board	14 (35.0)
**Age group (years)**	
20–29 years	3 (7.5)
30–39 years	10 (25.0)
40–49 years	9 (22.5)
50–59 years	9 (22.5)
≥60 years	9 (22.5)
**Location ^§^**	
Major urban area (100,000+ population)	11 (27.5)
Large urban area (30,000–99,000)	24 (60.0)
Medium urban area (10,000–29,999)	3 (7.5)
Small urban area (5000–9999)	2 (5.0)

* Multiple answers were allowed; hence, totals may amount to >40 and percentages may amount to >100%. ^†^ Multiple answers were allowed. However, not all healthcare professionals spoke two or more languages; hence, the total does not amount to 40. ^‡^ ‘Kaiāwhina’ is the over-arching term to describe non-regulated roles in the health and disability sector. Kaiāwhina support hauora (holistic wellbeing) outcomes. ^§^ Location is described according to the Statistics New Zealand Urban Rural indicator (https://www.stats.govt.nz/methods/functional-urban-areas-methodology-and-classification/) Accessed on 14 November 2025.

## Data Availability

Data are contained within the article.

## Glossary of Te Reo Māori Terms

Aotearoa	New Zealand
Hapū	Pregnant
Hui	Meeting, gathering
Iwi	Extended kinship group, tribe
Kaiāwhina	Helper, assistant, contributor, counsel, advocate
Kanohi ki te kanohi	Face to face, in person, in the flesh
Kaupapa Māori	Māori approach, Māori topic, Māori customary practice
Māmā	Mother, mum
Manaakitanga	Hospitality, kindness, generosity, support
Māori	Indigenous New Zealander, Indigenous person from Aotearoa New Zealand
Pākehā	Non-Māori of European descent, European New Zealander
Pēpi	Baby, infant
Tikanga	Customary practice, cultural practices, customs
Tino rangatiratanga	Self-determination, sovereignty, autonomy
Whakapapa	Genealogy, ancestry, genealogical links
Whakawhanaungatanga	Connect, establish relationships
Whānau ora	Wellbeing of whānau (extended family) as a group

## Abbreviations

The following abbreviation is used in this manuscript:

LMC	Lead Maternity Carer

## Appendix A. Interview Guide—Hapū Māmā

**Opening questions**

What do you think the main barriers are for people to be vaccinated in pregnancy?

What about for Māori hapū māmā?

*Participants were then given a short presentation showing them the vaccine maps and online booking tools available in Aotearoa.*

What you think of the intervention [map to vaccination services or online booking tool]

What do you like about the idea?

What don’t you like about it?

How would this positively or negatively motivate you to be vaccinated?

Prompt: Information delivery? (would it affect individuals’ appraisals of the risk of disease or the risk vaccination?)

How could this influence your intention to be vaccinated?

How could this influence your willingness to be vaccinated? Prompt: Confidence (would it affect individuals’ trust and confidence in the efficacy of the vaccine?)

What about those who are hesitant to vaccinate?

How would this intervention affect social processes regarding vaccination in pregnancy?

E.g. relationships with healthcare providers, other relationships—whānau?

Community acceptance of vaccination in pregnancy.

What about effect on ‘social norms’ of vaccinating in pregnancy?

Affect conversation about vaccination in pregnancy and the spread of information about vaccination?

How would it affect altruistic vaccination, i.e., the benefits of vaccinating to prevent spread of infection to their pēpi?

How would this intervention positively or negatively facilitate vaccination directly for you?

How would it remove barriers?

How could it support healthcare providers/vaccination workforce?

How would it facilitate healthcare providers to discuss vaccination?

How would it build on favorable intentions to vaccinate? (e.g., keep vaccination in peoples’ minds? reduce barriers?)

How could it shape behavior to vaccinate? (would it provide incentives?)

Would it work in your locality/community

Who should be responsible for promoting the intervention to Māori and Pacific hapū māmā?

How would you describe the potential usefulness, acceptability, and feasibility of the intervention?

What do you think other hapū māmā’s attitudes to the intervention would be?

How accessible would the intervention be to you and other Māori and Pacific hapū māmā?

How do you think this intervention would be used by you or other hapū māmā?

How would hapū māmā come to learn about the intervention?

How could this be made available to hapū māmā? emailed? Text? Online?

How should they be promoted—by a healthcare provider, e.g., midwife, GP, nurse, pharmacist? With whānau?

How can we make sure Māori and Pacific hapū māmā are seeing the intervention?

What are the strengths of this intervention? (Why would this work in your community?)

What are the weaknesses of the intervention? (What does it lack? Is there something missing?)

What opportunities are there for this intervention to work well?

Prompt: is it novel/a new idea?/Would it fill a niche?

Does it work well across the board? Will it support equity in vaccination provision?

Can you think of other ways it could help hapū māmā get vaccinated?

What would prevent this intervention from working well in your locality/community?

Why? Who or what group do you think it might not work for?

How would these interventions be supported by the community or other healthcare providers?

What do you think could help support primary care providers to use the videos in practice?

What do you think would prevent primary care providers using it in practice?

## Appendix B. Interview Guide—Key Stakeholders

**Opening questions**

What do you think the main barriers are for people to be vaccinated in pregnancy?

What about for Māori hapū māmā?

*Participants were then given a short presentation showing them the vaccine maps and online booking tools available in Aotearoa.*

What you think of the intervention [map to vaccination services or online booking tool]

What do you like about the idea?

What don’t you like about it?

How would this positively or negatively motivate Māori and Pacific pregnant people to be vaccinated?

Prompt: Information delivery? (would it affect individuals’ appraisals of the risk of disease or the risk vaccination?)

How could this influence their intention to be vaccinated?

How could this influence their willingness to be vaccinated? Prompt: Confidence (would it affect individuals’ trust and confidence in the efficacy of the vaccine?)

How could it influence the acceptability of vaccination for Māori and Pacific people?

What about those who are hesitant to vaccinate?

How would this intervention affect social processes regarding vaccination in pregnancy?

E.g. relationships with healthcare providers, other relationships—whānau?

Community acceptance of vaccination in pregnancy

What about effect on ‘social norms’ of vaccinating in pregnancy?

Affect conversation about vaccination in pregnancy and the spread of information about vaccination?

How would it affect altruistic vaccination, i.e., the benefits of vaccinating to prevent spread of infection to their pēpi?

How would this intervention positively or negatively facilitate vaccination directly for Māori and Pacific people?

How would it remove barriers?

How could it support healthcare providers/vaccination workforce?

How would it facilitate healthcare providers to discuss vaccination?

How would it build on favorable intentions to vaccinate? (e.g., keep vaccination in peoples’ minds? reduce barriers?)

How could it shape behavior to vaccinate? (would it provide incentives?)

Would it work in your locality/community

Who should be responsible for promoting the intervention to Māori and Pacific hapū māmā?

How would you describe the potential usefulness, acceptability, and feasibility of the intervention from the perspective of Māori and Pacific hapū māmā and their whānau?

What do you think healthcare providers and hapū māmā’s attitudes to the intervention would be?

How accessible would the intervention be to Māori and Pacific hapū māmā?

How do you think this intervention would be used by healthcare providers or hapū māmā?

How do you think this intervention could be used by healthcare providers or hapū māmā?

How could this be made available to hapū māmā? text/email/online?

How would hapū māmā come to learn about the intervention?

How should it be promoted—by a healthcare provider, e.g., midwife, GP, nurse, pharmacist? Whānau?

How can we make sure Māori and Pacific hapū māmā are seeing the intervention?

What are the strengths of this intervention? (Why would this work in your community?)

What are the weaknesses of the intervention? (What does it lack? Is there something missing?)

What opportunities are there for this intervention to work well?

Prompt: is it novel/ a new idea? / Would it fill a niche?

Does it work well across the board? Will it support equity in vaccination provision?

Can you think of other ways it could help hapū māmā get vaccinated?

What would prevent this intervention from working well in your locality/community?

Why?

(if applicable) How would these interventions be supported by the community or other healthcare providers?

What health service structures exist to support the intervention?

What do you think could help support primary care providers to use it in practice?

What do you think would prevent primary care providers using it in practice?

How should it be designed

How would the intervention need to be designed to meet the needs of Māori/Pacific hapū māmā?

What do you think could help support the intervention?

Prompt: What could be changed?

Prompt: Can you describe what support/resources would be needed for the interventions to work with Māori or Pacific women?

Is there anything we can be designed better to improve the interventions?

Prompt: How could we ensure that the interventions are effective for everyone?

Do you have anything else to add about interventions to improve maternal vaccination coverage?
